# Primary and Storage Batteries for Dental Purposes

**Published:** 1892-09

**Authors:** Jere. E. Stanton

**Affiliations:** Boston, Mass.


### PRIMARY AND STORAGE BATTERIES FOR DENTAL PURPOSES.[1]

BY JERE. E. STANTON, M.D., D.M.D., BOSTON, MASS.

In presenting this subject to you, and to the readers of the International Dental Journal, I am aware that it will provoke censure on account of the many failures that have been met with in the use of primary batteries. It is not my desire to induce any one to purchase an apparatus that will prove a source of annoyance to him.

---

[1] Read at a meeting of the Harvard Odontological Society, held at Boston, Mass., April 28, 1892.

Our Society has not had a paper on electrical apparatus, and, although I cannot treat the subject exhaustively, I feel sure my experience will prove of interest.

Electrical science has made such immense and rapid strides in the past few years that it is impossible for one not making it a business or profession to become anything like an expert, so I trust you will bear in mind that I am here to tell my experience with primary and storage batteries.

The first time I tried a battery for power was three years ago. For two years I tried various forms of bichromate of potash batteries, and all the motors that I could find that were made to run by a battery. A little more than a year ago I read an article on storage batteries that led me to think they might be used for dental purposes. After some investigation I learned that the capacity of a storage cell was limited, and many users said they were unreliable after a few months' active service, while others reported perfect satisfaction.

Having become interested in the subject of electrical storage, I continued to make inquiry whenever opportunity presented a chance of learning something about it, and finally concluded to go to Lowell, where a factory is in operation for the manufacture of storage batteries. There I learned the principle of their construction, why some failed, and others were successful.

It is well known that Planté devoted many years to the investigation of the storage capacity of different metals; that he finally decided that lead would deliver the greatest amount of electricity. So far as I know, no one has found a metal that will do as well.

Planté used plain lead-plates, but in a short time after he had published his researches, Faure produced a battery, more quickly made, and having a much larger storage capacity.

The sheet-lead of commerce has a slight coating of oxide on its surface; this oxide on the anode, or positive pole of the battery, is made a peroxide by the addition of oxygen; and the oxide coating on the cathode, or negative pole, is reduced to fine metallic lead, granular in form, by the action of hydrogen. By charging this battery, first in one direction, and then (after discharging it) charging it again in the opposite direction, and repeating this operation a number of times, it is found that the coatings grow thicker and thicker, till a point is reached beyond which no additional storage strength can be gained.

Faure conceived the idea of coating the plates with the red oxide of lead, made into a paste, holding it in place with felt placed between the plates, and placing them in acidulated water, as Planté did; by this means a much thicker coating could be placed upon the plates than could be done in a lifetime of charging and recharging. It is easy to see how by this means the capacity of the battery can be increased. The placing of felt, or any other material except vulcanite strips, or wood boiled in paraffine (for the purpose of keeping them in place, and to prevent them from touching), has been abandoned, and the paste of lead oxide is held in place by having the plates perforated or built up of lead-tape formed into small convolutions.

If a paste is used it is made by mixing one part of commercial sulphuric acid with two parts of water, letting the mixture stand till cold, then adding enough of the red oxide of lead to make a stiff paste; then, by means of a spatula, working it into the holes in the plates for the positive element of the battery. The same thing is done for the negative element, using litharge in the place of red lead, or there may be a combination of the litharge and red lead for both plates. After the paste has dried they are arranged alternately, with strips of some non-conductor not affected by acid between them and bolted firmly together; all of the positive plates are connected, making the positive pole, and the negative plates treated in the same way, making the negative pole. In very small batteries there are two positive and three negative plates; in the larger the plates are equal in number. The positive pole of the dynamo is now connected with the positive plates and the negative with the negative plates, and the plates are formed. This process takes in all about one hundred hours, and results in changing the red lead to peroxide, and the litharge to nearly metallic lead. The positive plates turn a very dark-brown or chocolate color, the negative a light-slate. The pasted plates are not reversed, but always charged in the same direction during the process of forming. After once being formed, both the pasted and the unpasted plates are charged and discharged in the same direction. It is not very difficult to make a battery of the unpasted type if one is so disposed, and such a battery will give good service in dental work for some time.

Before I decided to use a storage battery I made one with which to test the primary battery. I reasoned that if I could get a primary battery that would form and keep charged such a secondary battery, that it would do the work necessary for practical work. Having found a storage battery that I thought reliable, my next endeavor was to find some means of charging and keeping it charged.

A bichromate battery was out of the question, as that lasts but a short time on a short circuit, and the care of such a battery is unbearable. The common crow-foot, or such as is used on telegraph lines, is very dirty, gives only a small quantity of current, and unless a large number be used, would not keep the secondary battery charged sufficiently for practical use. I had read of a battery made in Boston that was rated to give about double the quantity of a common gravity battery, was clean, and needed but little attention. I looked it up, and found it a combination of the Daniel and crow-foot. The inventor, Mr. Gethins, said he felt confident that it would do the work; kindly put the volt-meter and ammeter into the circuit of some batteries that had been at work for some months, and after seeing the readings thought the experiment worth trying. Ordering enough to charge one cell, I at once made a small storage battery of plain lead-plates, and commenced forming them. The secondary cell was a very small one, not over two and a half inches square, but in about twenty-four hours there was power enough to make an eighth of a horse-power motor go several minutes, and in about two weeks it would run about twenty minutes, which was the capacity of the cell. This satisfied me that power enough could be obtained from a primary and storage battery to run a dental engine for practical use. The motor received my next attention. It was necessary to use as little power as possible, and a motor must be had that would run with the greatest economy. There are two ways that power is manifested in electricity, as in water,—viz., volume and pressure. If you have volume, you do not need pressure, and *vice versa.* The word volt means pressure, and ampère means quantity, in electrical nomenclature; and to give you an idea as to the relation of quantity and pressure, I will say a motor running on the Edison system takes one hundred and ten volts and about three-quarters of an ampère; a motor using the arc-light circuit would use twenty-five hundred volts and from seven to nine ampères. The motors in use on the street railroads are wound for six hundred volts and any number of ampères up to one hundred and fifty, and possibly more. I have seen the readings taken from the street roads, and they were from twenty to one hundred ampères. It is easily understood from what has been said that a motor can be wound for quantity or pressure, or both. In the use of a storage battery, if a motor is wound for volume or quantity, and the battery is a small one, it would take but a short time to exhaust the amount of electricity which the battery contained. If, however, it is wound for pressure, and not for quantity, the battery is drawn upon very slowly. Each storage cell gives a pressure of two volts, and is made to deliver about four hundred ampères. They can be much larger, but would be very heavy to handle. The cells which I use are thirty-five ampère-hour; that is, they will give one ampère thirty-five hours, or two ampères seventeen and a half hours, and so on, till about one-sixth of the capacity of the battery is reached, as it is not well to discharge it faster than one-sixth of its rated capacity; if this is done, the paste is in time forced out of the positive plates. Motors are wound for two volts and upward, but the number of ampères which they take is doubtful (I am speaking of battery motors) unless you have some means of measuring the amount as it is used. I found it necessary, therefore, to obtain an ammeter, and ascertain the amount of current different motors absorbed. Without detailing my work, I will say that I found a motor that required four volts and eight ampères loaded to its greatest capacity. This was within the range of my battery, and has proved more than satisfactory.

Having the storage battery and motor right, it only remained to get enough primary battery to keep the secondary fully charged. Having four volts' pressure in the secondary, it is readily understood that the primary must have a greater pressure in order to force the current into it, and the charging current should have a pressure of two and a half volts to each secondary cell; consequently, for two cells we must have at least five volts, and in the use of a battery I have found that six is more satisfactory, for the reason that the cells of primary do not give quite a volt each. In regard to the quantity, if an arc-light current is used it will be about nine ampères; but in using the incandescent current, from twenty to thirty ampères is generally used, according to the size of the accumulator, the time taken for charging being about seven hours.

Now, a battery gives a very small quantity compared to a dynamo, and it is found necessary to keep the accumulator in circuit all the time; therefore, we must have a battery that will work on a closed circuit without becoming exhausted; this we find in the common gravity, or, as it is called, crow-foot, and the Daniel cell. As I have explained, the former is very dirty, and gives only about one-third of an ampère, while the Daniel cell, if kept in closed circuit, causes the porous cup to become coated with copper, thereby reducing its efficiency in the matter of quantity. As yet no battery is perfect; an ideal one would run indefinitely without attention; we must take the one which approaches the nearest to what we desire, and in the Gethins we get very near it, giving, as it does, double the quantity, to say the least, of a gravity; is clean, and requires little attention. By using twelve cells of the Gethins battery, connecting them to give six volts, or two in series, we get nearly six volts, and from one to one and a half ampères, enough to keep the storage battery charged (if kept in circuit all the time) and run the engine, mallet, and mouth-lamp, if desired. I have had my motor running for two years constantly, without its once failing, and the batteries are as good as when first put in. There is almost nothing to do to the accumulators; once in six months, perhaps, a little water has to be added to make up for evaporation,—that is all. The primary needs a little more care. If the room in which they are kept is warm, it is necessary to put, once a week, a little water in the porous cups, and as the copper sulphate is exhausted at the end of two months, that has to be renewed at that time.

If the entire battery is renewed at one time, it takes about an hour to do it. I find it most convenient to replenish two or four cells at a time, and in a day or two, when I have a few moments, treat others; in this manner the battery is kept in good condition, and the time spent on it is, to me, not noticed.

The space occupied is small; there are no fumes, absolutely no odor, and nothing about it to stain the hands. It seems to me necessary to have the battery near plenty of water, and where it would be under frequent inspection; by having it thus arranged one would take better care of it than if it were in some secluded place, likely to be forgotten so long as the motor would go. It is necessary to bear in mind the fact that the charging of storage batteries is the hardest work a battery is called upon to do; we cannot possibly get power without the consumption or the decomposition of something, and it is observed that the zincs are decomposed and waste, leaving a residuum in the porous cups which has to be removed. Also, the water in the cups becomes saturated with zinc, and the battery does not work quite as well in consequence; for this reason I find it better to occasionally wash out the porous cups with cold water; this can be done at odd moments as the occasion demands. My own batteries are in a side room, about fifteen feet from the chair. If it is necessary to have them at a distance, I think the storage batteries should be near the motor, using a large size of wire not less than No. 12, better 10, from the primary. By so doing there is no loss from the storage to the motor. I also keep a tangent galvanometer, which any one can make, in the circuit, from the primary to the secondary; this shows at a glance if the battery is in a good condition. In making the galvanometer it is necessary to use a large wire or strip of copper, so the resistance shall not be greater than the conducting wires. All connections should be perfect; if anything is wrong it is more likely to be the connections than anything else. The circulars which are sent with the batteries give directions as to their care, which is embodied in what I have said.

Since the storage battery has become an undoubted success, and it has been learned that they can be charged by means of a primary battery, there have been a great many batteries put upon the market for the purpose of charging them, but they are failures, and to-day, so far as I can learn, the Gethins is the only one that stands for the work for any length of time without giving out. Others show well for a few days, but rapidly fail and have to be renewed, as does the bichromate-of-potash battery. The law of compensation holds good here as elsewhere; if there is developed a large amount of current, it is at the expense of something, and that something is in this case the battery elements or solution. While the Gethins does not give a great quantity of current, it gives what it does produce for a long time, and without attention. This can be easily stored and used as wanted.

It is also better to connect two in multiple than have the twelve in series, as it is found this arrangement prevents the rapid consumption of the zincs, which takes place where they are in series. The primary must be a little in excess of the storage, but not too much. If it is, the primary is more nearly on a short circuit, and that means rapid consumption of the zincs. I have been asked why not have a larger storage battery to give a larger quantity in number of hours which the battery would run the motor; the reason is this: the thirty-five ampère-hour cell is large enough to run the engine all one wants to in a day, and if it were larger the primary would not fully charge it. If a storage battery is kept partly discharged all the time the plates are injured; in fact, in time ruined; therefore it is necessary to have the accumulator small enough for the primary battery to keep it fully charged, which is the best condition for a secondary cell or cells.

It may be of interest to some to know why I use the Sorley storage battery. I think it the only one on the market that is reliable, and, from what I have seen of others, the only one in which the plates will not warp, and from which the paste will not fall out. The trouble with accumulators has been from short circuits in the battery itself, caused by the warping of the plates and the falling out of the paste; the plug getting between the plates causing local action and the exhausting of the battery. I have seen a number of such cases, the battery doing good work for a time, then gradually failing, and at last becoming worthless. Such a battery in the hands of one not posted on the work of accumulators would probably be cast aside and pronounced useless, or the owner would be at quite an expense for repairs, and in the course of time the same thing would be repeated. In making one from lead-plates great care must be taken to arrange the plates so they will not warp or become short-circuited by the sediment which falls to the bottom of the jar in all kinds of storage batteries. You will see by the samples which I have brought that the plates do not touch the jar at the bottom, and the powder that comes out cannot lodge anywhere, but must fall to the bottom. Another is what is called sulphating, or the formation of the higher sulphates on the plates; this is, I think, more likely to take place where the secondary battery is charged with a primary than where a dynamo is used, and is indicated by the formation of white spots on the plates. I find the best way to remove such formation is to rub it off with a thin stick, on the end of which is wound a piece of string that will easily pass between the plates.

The elements are lifted out of the acid and washed, then the white deposit is easily rubbed off; it can be removed by overcharging with a strong current, but I have not succeeded in taking it off with a battery. Any considerable quantity of this sulphate should not be allowed to form, for, once started, it will grow like a fungus, and in time reduce the efficiency of the battery very much. As a matter of fact, I think that the accumulators are always overcharged at the stations, a practice which keeps them in good condition; this is not easily done with a battery, unless more battery is used than I employ. The voltage is enough, but there would need to be three in series instead of two, making eighteen in all. When a battery is fully charged it will boil, and by putting the ear to it the liberation of gas can be distinctly heard; if the elements are in glass this process can be seen. The connection between the cells should be particularly good, and if the cells are moved, it not infrequently happens that they are broken, and the motor will not go, on account of this slight disturbance. It occasionally happens that the power is not quite up to standard; the batteries seem all right, and you wonder what the matter is; at such times it is well to see if the brushes on the motor are in the right place; as they become worn they wear back from the most effective point, and should be moved forward to their original position on the commutator, which is easily found by making connection with the slowest speed, and moving the brushes to that point where the motor runs the fastest. All bearings should be kept well oiled, as there is no surplus power to be wasted in unnecessary friction, and it is much better to have rubber bands on all pulleys; this will allow the belt to be run very loose, at the same time giving full power. There is no possible means of getting or giving a shock; the speed can be made very much slower than any motor that I know of; may run from a street-wire (a very desirable feature), and the power is not so great that there is any harm done if anything gets caught. It is always ready; the power is more than enough for anything one can do with the engine; in fact, will run the lathe, if the stones are not over one and three-quarters of an inch in diameter; and the cost of running is about seventy-five cents a month. I have placed my motor and rheostat on one base, taking up no more room and weighing no more than the engine. It can be moved to any place by having the flexible wire long enough, and when not in use is pushed to one side, same as the engine. I do not claim that it is better than the current from the street, but I do think it is just as good, barring the care of the battery, which to me is very little trouble. From what has been said do not for a moment think I desire to urge any one to purchase this outfit, because I have obtained so much satisfaction from it. I know perfectly well that what one man finds a convenience, another rates as a nuisance; but if one will spend as much time on the battery as in keeping other instruments in order, the labor of dental operations will be greatly lessened.

