# Correction

**Published:** 1892-10

**Authors:** 


### CORRECTION.

THE date on the thirteenth line from the bottom on page 651 of Dr. Sudduth's article in the last number (September) should read 1889–90 instead of 1890–91.

